# The Neurobiology Shaping Affective Touch: Expectation, Motivation, and Meaning in the Multisensory Context

**DOI:** 10.3389/fpsyg.2015.01986

**Published:** 2016-01-06

**Authors:** Dan-Mikael Ellingsen, Siri Leknes, Guro Løseth, Johan Wessberg, Håkan Olausson

**Affiliations:** ^1^MGH/HST Athinoula A. Martinos Center for Biomedical Imaging, Massachusetts General Hospital, Harvard Medical SchoolBoston, MA, USA; ^2^Department of Psychology, University of OsloOslo, Norway; ^3^Institute of Neuroscience and Physiology, University of GothenburgGothenburg, Sweden; ^4^Department of Clinical and Experimental Medicine, Linköping UniversityLinköping, Sweden

**Keywords:** touch, top–down modulation, hedonics, oxytocin, opioids, social processing, placebo effect

## Abstract

Inter-individual touch can be a desirable reward that can both relieve negative affect and evoke strong feelings of pleasure. However, if other sensory cues indicate it is undesirable to interact with the toucher, the affective experience of the same touch may be flipped to disgust. While a broad literature has addressed, on one hand the neurophysiological basis of ascending touch pathways, and on the other hand the central neurochemistry involved in touch behaviors, investigations of how external context and internal state shapes the hedonic value of touch have only recently emerged. Here, we review the psychological and neurobiological mechanisms responsible for the integration of tactile “bottom–up” stimuli and “top–down” information into affective touch experiences. We highlight the reciprocal influences between gentle touch and contextual information, and consider how, and at which levels of neural processing, top-down influences may modulate ascending touch signals. Finally, we discuss the central neurochemistry, specifically the μ-opioids and oxytocin systems, involved in affective touch processing, and how the functions of these neurotransmitters largely depend on the context and motivational state of the individual.

## Introduction

Inter-individual touch is frequently used to communicate positive messages, like reassurance, comfort, sympathy, and support (Hertenstein et al., 2006b). For the recipient, touch from another person can be soothing (Feldman et al., 2010b; Fairhurst et al., 2014), give rise to pleasurable feelings (Löken et al., 2009; Morrison et al., 2010), and potentially suppress pain and negative emotion (Coan et al., 2006; Liljencrantz et al., 2012; Mancini et al., 2014, 2015). On the other hand the hedonic experience of touch can be flipped from pleasure to displeasure if the perceived intentions or the identity of the toucher does not match the preferences of the recipient of touch (Gazzola et al., 2012).

The hedonic value of touch, the pleasantness or unpleasantness, is intrinsically related to the physical characteristics of tactile stimuli, like softness (Rolls et al., 2003), temperature (Schepers and Ringkamp, 2009; Ackerley et al., 2014), force and velocity (Löken et al., 2009). However, as in other sensory modalities, the signals from the peripheral receptors are processed and modulated by several “top–down” mechanisms before the subjective experience of touch arises in the brain (Kveraga et al., 2007; Ellingsen, 2014). First, sensory information enters subjective awareness through the gate of attention (Johansen-Berg et al., 2000) – presuming you are sitting down right now, you might not be aware of the physical pressure of the chair pressing against your skin until this very moment when your attention is directed toward this stimulus (Schubert et al., 2008). Second, when sensory signals do gain access to awareness, the resulting sensation is influenced by the brain’s pre-existing models, or predictions, of what these sensory signals mean, which are shaped by learning (Knill and Richard, 1996; Kersten et al., 2004; Schmack et al., 2013). Third, other available cues carrying information about the importance, relevance and affective valence of this sensation, weigh in. For a given affective touch stimulus, contextual information such as visual or auditory cues about the toucher (Macaluso and Driver, 2001; Taylor-Clarke et al., 2002), and internal motivational state or mood (Kalaska, 1994; Montoya and Sitges, 2006; Triscoli et al., 2014; Løseth et al., in press), is essential for deciding how important this particular touch is (how much attention should be paid to it), how desirable it is (positive or negative), and how to respond behaviorally.

Most of the research on affiliative touch has been done from the vantage point of the touch stimulus itself, e.g. the neurobiology of mechanoreceptive skin receptors and the ascending touch pathways (Vallbo et al., 1993; Wessberg et al., 2003; Vrontou et al., 2013), observational studies of animals’ engagement in specific touch behaviors (Harlow and Zimmermann, 1959; Dunbar, 1991; Alberts, 2007), or human psychophysical (Loken et al., 2011; Ackerley et al., 2014; Fairhurst et al., 2014) and neuroimaging (Rolls et al., 2003; Olausson et al., 2008; McGlone et al., 2012; Bjornsdotter et al., 2014; Kaiser et al., 2015) studies assessing the sensations and brain activity in respect to different touch stimuli. Much less is known about the neurobiological processes whereby top-down factors – cross-sensory, cognitive, and affective information – shape touch signals.

Here, we review the neural circuitry and neurochemistry underpinning top-down modulation of affective touch, and suggest how the brain integrates sensory and prior information into affective touch sensations. First, we discuss how context modulates the meaning and in turn the hedonic value of touch, and how this shapes both the affective experience and the behavioral consequences. We will then review the central neurochemistry, primarily μ-opioids and oxytocin, underpinning the seemingly opposite stimulatory and soothing effects of touch, and propose how these outcomes are highly dependent on the individual’s affective-motivational state.

## Reciprocal Influences of Touch and Context

Much of human behavior is geared toward seeking pleasant experiences, while avoiding unnecessary painful, or aversive experiences. Hedonic valuation of sensation guides decisions about which behaviors to engage in and which to avoid, rendering hedonic processing essential to survival (For review, see Berridge and Kringelbach, 2015). In order to be useful, however, these systems need to take into account the individual’s short-term and long-term needs. While high-calorie food is usually thought of as a desirable reward, it loses its utility and ceases to be pleasurable upon satiety (Small et al., 2001). Similarly, the utility and consequently the hedonic experience of interpersonal touch largely depends on the context and internal needs and motivational goals.

### Modulation of Touch Experience by Context and Internal State

When being touched by another individual, inferences about the identity, physical characteristics, and the intentions of the toucher, conveyed through visual and auditory stimuli, gives useful information about the importance of the touch and how preferable it is (Suvilehto et al., 2015). This can dramatically shape both the hedonic experience and the behavioral response (e.g., approach or withdraw).

**FIGURE 1:**
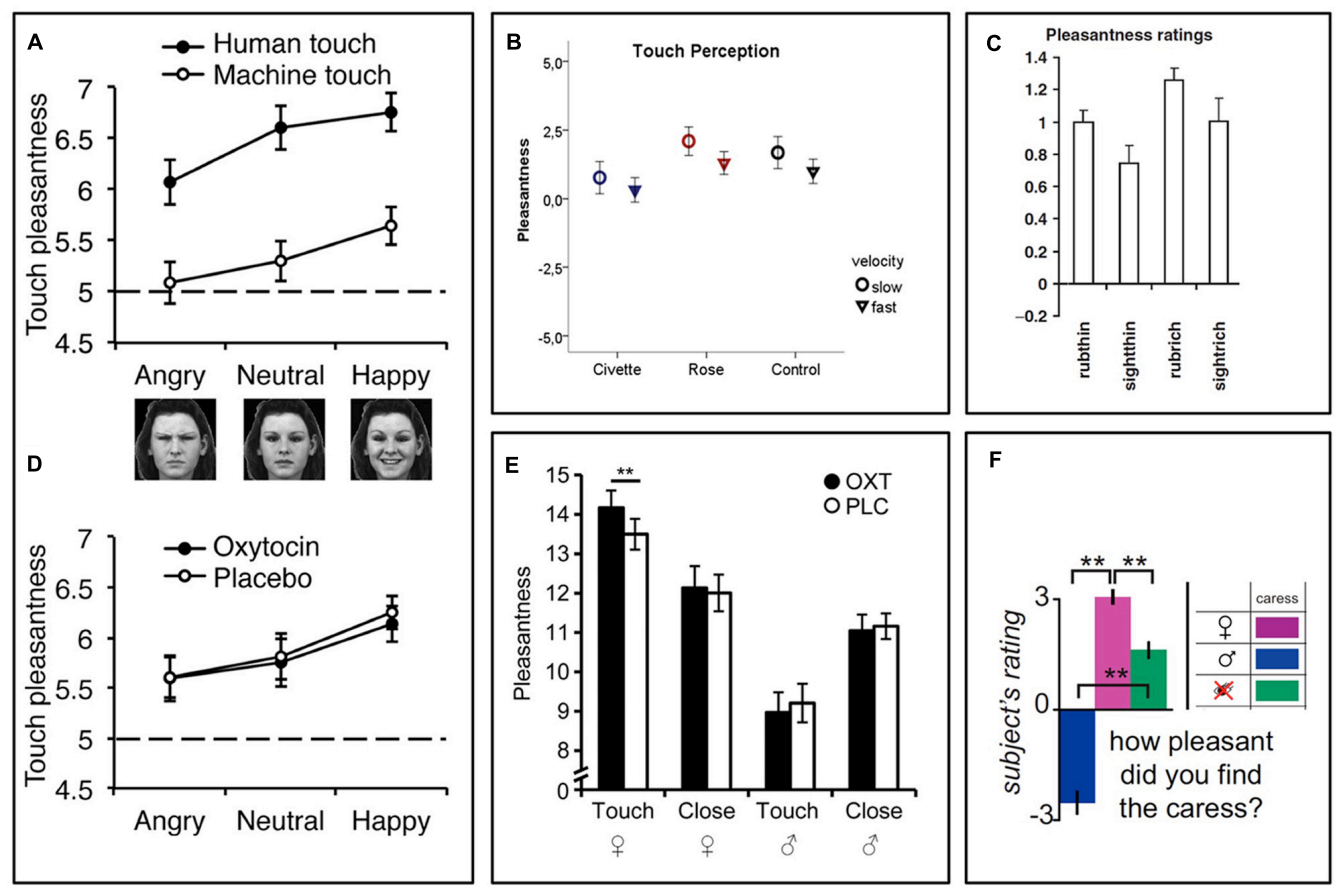
**Contextual modulation of touch pleasantness during identical tactile stimuli. (A)** Touch pleasantness of both gentle stroking (human) touch and equally intense vibratory (machine) touch is highest during concomitant presentation of smiling faces and lowest during presentation of frowning faces (Ellingsen et al., 2014). **(B)** In a similar fashion, touch pleasantness is highest during concomitant presentation of pleasant (rose) odors and lowest during presentation of disgusting (civette) odors (Croy et al., 2014). **(C)** People find the gently rubbing of a skin cream more pleasant when being told it is a “rich moisturizing cream” (rubrich) relative to a “basic cream” (rubthin) (McCabe et al., 2008). **(D)** While one study found no effect of intranasal oxytocin on touch pleasantness (Ellingsen et al., 2014), another study **(E)** found that oxytocin increased touch pleasantness in heterosexual men when they believed they were being sensually caressed by a woman, but not when they believed the caresser was a man (Scheele et al., 2014). **(E–F)** Caresses are more pleasant when the caresser is believed to be a woman relative to a man (Gazzola et al., 2012; Scheele et al., 2014). Figure adapted from (McCabe et al., 2008; Gazzola et al., 2012; Ellingsen et al., 2014; Croy et al., 2014; Scheele et al., 2014). ^∗∗^*p* < 0.01.

In two similar experimental studies of interpersonal touch, the recipient’s beliefs about the toucher affected the pleasantness of gentle sensual caresses (**Figure 1**; Gazzola et al., 2012; Scheele et al., 2014). The study participants, who were all heterosexual men, rated experimentally applied sensual caresses as pleasant when they were lead to believe, via a visual cue, that they were being caressed by a female experimenter, but unpleasant when the cue indicated a male experimenter. In reality the same female experimenter, who was blinded to the cues, did all the caresses. For the study participants, the visual information about the sex and appearance of the believed toucher changed the meaning and desirability of the touch, which in turn impacted the hedonic touch experience – touch by an attractive female felt better.

Using a different design, we recently showed that the visual presentation of faces with emotional expressions affected the pleasantness of concomitant touch stimuli. Study participants rated touch as most pleasant when combined with a photograph of a smiling face and least pleasant when combined with a frowning face (**Figure 1A**, Ellingsen et al., 2014). Interestingly, this effect was seen even though participants were fully aware that the person they saw in front of them was not the person touching them. This suggests that affective cross-sensory stimuli that are time-locked with the touch, but seemingly non-informative (i.e., does not provide any specific information about the value of the touch stimulus), can still influence the hedonic impact of this touch. Along the same lines, a recent experiment showed that disgusting odors presented simultaneously with a gentle stroking touch reduced the pleasantness of this touch (**Figure 1B**). Again, the participants were fully aware that the stimuli originated from independent sources (Croy et al., 2014). Instead of carrying information about the value of the touch itself, these effects may appear as a result of a shift in affective or motivational state, which in turn change the hedonic impact of touch, perhaps similar to affective priming (Winkielman et al., 2005; Schwarz, 2012). This bears similarity to the tendency of unpleasant and pleasant sensory events to exert immediate reciprocal inhibitory effects. For example, pleasant images, odors, music, and food can reduce pain (For review, see Leknes and Tracey, 2008). On the other hand, pain and negative affect can reduce the capacity for pleasure, as demonstrated by the strong comorbidity between chronic pain, depression, and anhedonia (i.e., a lack of capacity for pleasure; Pizzagalli et al., 2008; Elvemo et al., 2015; Romer Thomsen et al., 2015). However, there are also cases where pain can *enhance* the pleasure of reward (Carstens et al., 2002; Rozin et al., 2013; Bastian et al., 2014; Leknes and Bastian, 2014), highlighting that the relationship between pleasure and displeasure is not one of a simple, universal mutual inhibition, but rather involves a complex integrative process weighing the importance of contextual cues.

A widespread notion is that sensations in general are shaped by inferences about the relative importance, or utility, of sensory signals (Cabanac, 1971; Tindell et al., 2006) – how useful or relevant these are in relation to the organisms’ goals, which are often ultimately related to survival, well-being, and procreation. The motivation-decision model of pain was put forward to explain the often-dramatic variability in pain experience due to the individual’s internal motivational state (Fields, 2006, 2007). This framework describes brain mechanisms that reduce or increase the hedonic impact of nociceptive events based on their relative importance at the given time. The model was initially developed to explain modulation of pain, but the basic idea is applicable to affective touch, as well as other sensory events that fall within a reward-punishment continuum. The model postulates that, as a result of an unconscious decision-making process, any concurrent or impending event deemed more important to the individual than a pain stimulus should suppress the hedonic impact of this pain. The event of superior importance may be a greater threat or a potential reward. Likewise, anything judged as more important than an impending reward – for example a threat or a bigger reward – should suppress the hedonic impact and motivation for this reward (Fields, 2011).

Like pain, touch stimuli usually happen in a multisensory context. As a facet of this, the occurrence of touch can affect the experience of non-touch stimuli – just as other sensory stimuli co-occurring with touch can affect touch experience (Taylor-Clarke et al., 2002; Calvert and Thesen, 2004).

### Behavioral and Cognitive Effects of Touch

Being touched by another human being can evoke powerful emotions. People are remarkably accurate in detecting a wide range of emotional messages, even when these are communicated exclusively through touch (Hertenstein et al., 2006a). A series of observational studies has showed that brief, casual touch from strangers can have positive behavioral effects in people, and even make them more generous. Restaurant diners tip more if the waitress casually touches them when returning their change (Crusco and Wetzel, 1984), and people are more satisfied with a library visit if the librarian casually touches their hand (Fisher et al., 1976). Similar studies report that when casually touched, people are more likely to return money left in a public phone (Kleinke, 1977), spend money in a supermarket (Hornik, 1992), rate salespeople at car showrooms more positively (Erceau and Guguen, 2007), or give away cigarettes (Joule and Gueguen, 2007). There are also studies suggesting positive health effects of touch in therapeutic relationships (Whitcher and Fisher, 1979; Eaton et al., 1986; Monroe, 2009), and within romantic couples (Grewen et al., 2003; Ditzen et al., 2007).

In most such studies, however, touch formed part of an affectively congruent situation. Less is known about the effects of, and appraisal of, touch in contexts where other available information is affectively incongruent, such as being casually touched by someone expressing anger. On the interplay between touch and concomitant non-touch signals, touch has been proposed to intensify the emotional display of other senses (Knapp and Hall, 1997; Hertenstein et al., 2006a). Touch ultimately means that someone – or something – is making physical contact, for better or for worse, which often calls for immediate action. A potential intensifying effect of touch on other sensory signals might therefore facilitate a rapid decision on whether the toucher is a friend or a foe, which is essential when this person is close.

We recently found that gentle touch from another human shaped social impressions of visually presented faces differentially depending on the emotional expression of the face (Ellingsen et al., 2014). Whereas concomitant human touch made innocuous neutral and smiling faces seem more attractive and friendly, it made angry faces seem less attractive and friendly, relative to equally intense touch from a device. This effect was potentiated by intranasal administration of an oxytocin receptor agonist (Ellingsen et al., 2014) (See below for more on oxytocin).

## Brain Mechanisms Underpinning Top–Down Modulation of Touch

To understand how affective touch experiences are created in the brain, it is useful to examine first, how touch stimuli are transmitted from the periphery to the brain, and second, how these signals are modified by and integrated with top-down information.

The processing of touch starts with the activation of mechanoreceptive afferents in the skin, such as fast-conducting, myelinated A-beta or slow-conducting, unmyelinated C-tactile (CT) afferents (Olausson et al., 2010; McGlone et al., 2014). While A-beta afferents respond to a wide variety of touch stimuli, CT afferents may be more specifically tuned to respond to stimuli slowly moving over the skin, like a caress, and their firing rates in the peripheral afferent correspond closely to touch pleasantness (Löken et al., 2009). Moreover, CTs activate most vigorously in response to touch stimuli that are close to skin-temperature, but less to colder or warmer stimuli, which again corresponds closely to pleasantness ratings (Ackerley et al., 2014).

Less is known about the relationship between CT signaling and positive affect during different contexts or motivational-affective states (but see Croy et al., 2014). Notably, however, recent studies suggest that CT afferents may play a role in the tactile “hedonic flip” following injury or inflammation of the skin (such as tactile hypersensitivity and allodynia), whereby light gentle touch becomes less pleasant or even painful (Liljencrantz and Olausson, 2014). Recent animal studies have found that inflammation-induced hypersensitivity is reduced in mice whose transmission of C-low-threshold mechanoreceptive afferents (equivalent to CTs in humans) has been genetically knocked out (Seal et al., 2009; Lou et al., 2013). In humans, experimentally induced allodynia-like pain, provoked by light touch to the skin overlaying an aching muscle, persists after functional compression blockage of myelinated skin afferents (Nagi et al., 2011). Other studies have found that, using the experimental heat/capcaicin model of allodynia, light CT-optimal touch (3 cm/s velocity) to the skin adjacent to the sensitized area was more unpleasant than CT-suboptimal touch (30 cm/s), reversing the relationship between velocity and pleasantness seen under healthy conditions (Liljencrantz et al., 2014). This is consistent with the view that a potential antinociceptive role of CT is disrupted, or that CT afferents may even signal negative affect, during injury or inflammation of the skin (Liljencrantz and Olausson, 2014). Thus, it is possible that during such physiological “threat conditions” (Fields, 2006; Porges, 2007), a state-induced shift in the function of CT afferents may contribute to the motivation to protect and care for a wounded limb.

Given the strong contextual influences on touch pleasantness, it is unknown whether there are qualities of certain touch stimuli that *inherently carry* a positive hedonic value (i.e., are pleasant or give rise to positive affect), or whether the hedonic value of touch is *always dependent* on other contextual or internal factors (Ellingsen et al., 2015).

A-beta afferents from the upper and lower extremities terminate in the cuneate and gracile nuclei of the dorsal column (Perl et al., 1962; Petit and Burgess, 1968), where they synapse onto neurons that transmit to the ventral posteriolateral nuclei of the thalamus. C-tactile afferents likely take a different route to the brain, through the spinothalamic tract (Andrew, 2010). From the thalamus, touch signals are relayed to cortical sensory processing areas such as the insular (Olausson et al., 2002; Bjornsdotter et al., 2009) and primary and secondary somatosensory areas (McGlone et al., 2002; Gazzola et al., 2012) as well as to other higher-order areas such as the prefrontal, orbitofrontal, anterior cingulate cortices, and the superior temporal sulcus (Francis et al., 1999; Lindgren et al., 2012; McGlone et al., 2012; Gordon et al., 2013; Scheele et al., 2014). There is also evidence that subcortical areas such as ventral striatum and amygdala, which are key structures in the processing of affect and motivation in general, are implicated in the processing of affective touch (Ellingsen et al., 2013; Perini et al., 2015).

Although the subjective experience of pleasant touch is thought to arise from cortical activation, it is not clear how other information, such as visual contextual information or memory, modulates the sensory signals. Does such information target the neural systems that generate pleasure or displeasure, e.g., hedonic hot and cold spots (Pecina and Berridge, 2005; Ho and Berridge, 2013; Castro and Berridge, 2014), or does it also modulate ascending sensory signaling? If so, at what levels does this modulation take place? Evidence from different fields indicates that top-down influences can modulate sensory signals at early stages of sensory processing. Focused auditory attention in humans can modulate signaling in the auditory sensory cortex as early as 20 ms post stimulus (Woldorff et al., 1993). Moreover, visual spatial attention can modulate pre-cortical signals in the lateral geniculate nucleus of the thalamus, the first relay between the retina and the cortex (McAlonan et al., 2008). It is well documented that ascending nociceptive neurons in the spinal dorsal horn are modulated by signaling descending from the brain (Wall, 1967; Woolf, 2011). The periaqueductal gray (PAG) in the midbrain controls incoming nociceptive signals indirectly through the rostroventral medulla (RVM;Millan, 2002; Fields, 2004). Neurons in the RVM project to the spinal dorsal horn, with inhibitory or excitatory effects on nociceptive transmission (Urban and Gebhart, 1999; Neubert et al., 2004). The PAG receives direct input from the limbic structures amygdala and ventral striatum, and from the prefrontal cortex, constituting a descending pathway by which affective or cognitive information can influence ascending sensory information already at the spinal dorsal horn (Fields, 2004).

Modulation of innocuous touch is less studied, especially in humans. A few studies on somatosensory evoked potentials (SEPs) have shed light on what levels of sensory processing may be modulated by top-down influences. One study found SEP differences as early as 50 ms post-stimulus when study participants were attending to, relative to not attending to, tactile stimulation of the index finger (Schubert et al., 2008). Another study found SEP differences when people were led to expect a more intense tactile stimulus, relative to an expected low-intensity tactile stimulus (Fiorio et al., 2012). These studies, in which the stimuli were identical regardless of attention or expectation, suggest that somatosensory processing can be modified by top-down processing at least as early as the primary somatosensory area.

In rodents, there is electrophysiological evidence that corticofugal projections, originating from the primary somatosensory area (SI), modulate innocuous touch signals in the cuneate and gracile nuclei of the dorsal column – the earliest relay stages for many low-threshold mechanoreceptive afferents (Nunez and Malmierca, 2007). Further, branches of low-threshold mechanoreceptors synapse at the segmental level in the spinal dorsal horn, but it is not known if central cognitive or affective information can alter touch processing at this level (Abraira and Ginty, 2013).

### Brain Mechanisms Underpinning Contextual Modulation of Affective Touch

Modulation of hedonic sensations by context, expectations, attention, and mood, can sometimes alter widespread sensory processing in the brain (Small et al., 2001; Wager et al., 2004; de Araujo et al., 2005; Petrovic et al., 2005; Nitschke et al., 2006; Tracey and Mantyh, 2007; Berna et al., 2010; Knudsen et al., 2011; Woods et al., 2011; Amanzio et al., 2013). Such modulations have been widely studied in paradigms evoking placebo effects, i.e., beneficial effects from clinical treatment due to patients’ or study participants’ positive expectations or appraised contextual meaning, rather than the active ingredient of the treatment itself (Schedlowski et al., 2015; Wager and Atlas, 2015). In these experiments, contextual cues are often manipulated to alter the subjects’ expectations of the effects of the treatment, which can be an inactive substance or procedure. Thus, one can study how an unpleasant sensation or symptom, such as pain, changes across different contexts. A series of functional neuroimaging studies indicate that placebo improvement is often underpinned by modulation of neural circuitry that traditionally are considered pathways for bottom-up, ascending sensory signals (Buchel et al., 2014). For example, placebo-induced reduction of pain is often associated with widespread reductions of somatosensory processing in thalamus, insula, primary and secondary somatosensory areas, and dorsal anterior cingulate cortex (ACC; Price et al., 2007; Eippert et al., 2009a; Lu et al., 2010; Amanzio et al., 2013). Moreover, some studies suggest that nociceptive processing in the spinal cord can be modified by expectations of pain relief (Eippert et al., 2009b) or pain worsening (Geuter and Buchel, 2013). Increased activity in a set of brain regions collectively involved in cognition, valuation, and affective processing, consisting of ventromedial (vmPFC) and dorsolateral (dlPFC) prefrontal cortex, orbitofrontal cortex (OFC), anterior insula, ventral striatum, amygdala and the midbrain, is often observed in placebo studies (Petrovic et al., 2002; Wager et al., 2004; Zubieta et al., 2005; Scott et al., 2007, 2008; Watson et al., 2009; Geuter et al., 2013; Pecina et al., 2013; Bingel and Placebo Competence Team, 2014; Hashmi et al., 2014; Kessner et al., 2014; Wrobel et al., 2014; Sevel et al., 2015), and is thought to be responsible for the suppression of pain processing. The functional architecture of modulatory networks for placebo responsiveness has yet to be disentangled, but these regions play central roles in hedonic valuation more generally, in the monitoring and updating of expectation, and the integration of available relevant information (Craig, 2009; McDannald et al., 2011; Schoenbaum et al., 2011; Roy et al., 2012; Lebreton et al., 2015; Lindquist et al., 2015).

**FIGURE 2:**
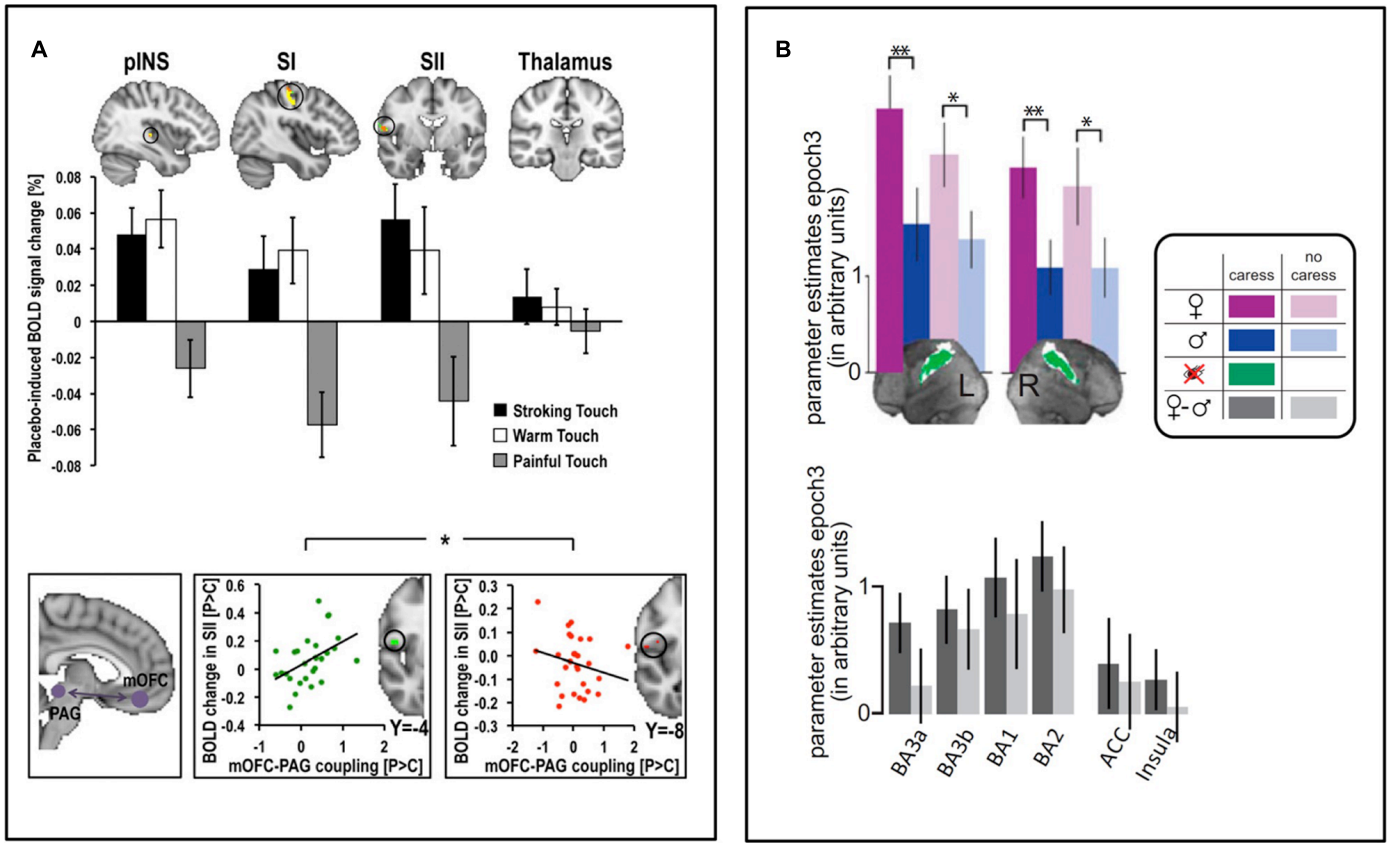
**Contextual modulation of brain to affective touch. (A)** After self-administrating a (placebo) nasal spray believed to have beneficial effects on gentle touch and pain perception, placebo-induced increases in touch pleasantness and reductions in pain unpleasantness were underpinned by respective increases and decreases in somatosensory processing of pleasant touch and pain (top). The individual magnitude of this somatosensory modulation was associated with the degree to which placebo treatment increased the functional connectivity between the medial OFC (mOFC) and PAG (bottom, left), an important pathway for pain modulation – those with the strongest increase in mOFC-PAG connectivity had the strongest somatosensory increases to pleasant touch (bottom, middle) and the strongest somatosensory decreases to pain stimuli (bottom, right) (Ellingsen et al., 2013). **(B)** In heterosexual men, SI activity during gentle caresses was larger when they believed a woman, relative to a man, performed the caress. The same pattern was seen across different sub-regions of SI, as well as (non-significantly) in the ACC and insula (bottom) (Gazzola et al., 2012). Figure adapted from (Gazzola et al., 2012; Ellingsen et al., 2013). ^∗^*p* < 0.05, ^∗∗^*p* < 0.01.

There are relatively few investigations of the brain mechanisms underpinning contextual modulation of affective touch. A handful of studies have used functional Magnetic Resonance Imaging (fMRI) to investigate brain activity responses to the same touch gentle stimulus during different contexts. We recently investigated whether placebo improvement of touch pleasantness (hyperhedonia) involves a modulation in somatosensory processing circuitry and whether this is related to activation of a prefrontal–subcortical modulatory neural circuit, similarly to that observed in placebo analgesia (Ellingsen et al., 2013). We suggested to a group of healthy volunteers that a nasal spray would increase both the pleasantness of gentle touch and reduce the unpleasantness of pain. After self-administration of a placebo nasal spray, which the participants were lead to believe would improve the affective aspects of both gentle touch and pain sensations, they found touch more pleasant and pain less unpleasant. While fMRI recordings during pain stimuli indicated decreased somatosensory processing, recordings during gentle touch stimuli showed instead increased activity in somatosensory areas (SI, SII, and the posterior insula). Those participants who showed the strongest placebo hyperhedonia and analgesia, also had the strongest placebo-induced activity increase in vmPFC, Nucleus Accumbens, amygdala, and brainstem regions. Furthermore, the magnitude of this activity increase was related to the modulation of somatosensory circuitry. Specifically, those with the strongest placebo increase in functional coupling between vmPFC and PAG also had the strongest hyperhedonic increases and analgesic decreases in somatosensory areas (**Figure 2A**), consistent with previous findings for placebo analgesia (Wager et al., 2007; Eippert et al., 2009a). Another fMRI study investigated the modulation of touch pleasantness during the application of a skin cream, by the visual presentation of labels saying either “rich moisturizing cream” or “basic cream” (**Figure 1C**, McCabe et al., 2008). Although it was always the same cream, participants reported the application of the rich cream as richer and more pleasant. This improvement in hedonics was associated with increased activations in the ventral striatum, pregenual ACC (pgACC), SI/SII, and the parietal area 7. One study found that when manipulating study participants’ beliefs about the gender identity of the toucher, touch pleasantness of “female caresses” increased, along with activation increases in SI and the OFC (**Figures 1E** and **2B**; Gazzola et al., 2012). Using a similar design (**Figure 1D**; Scheele et al., 2014) partly replicated these results, showing increased activation in the SI, as well as the caudate, when participants believed the caresser was female. Moreover, the intranasal administration of an oxytocin receptor agonist further increased the touch pleasantness of the “female caresses”, which was underpinned by activation increases in the anterior insula, pgACC, and precuneus.

It has not yet been demonstrated whether the modulation of pleasurable touch, like pain, involves descending modulation of cutaneous afferents in the spinal cord, perhaps via the RVM. Nevertheless, these findings suggest that, like negative hedonic feelings such as pain, psychological modulation of pleasant sensations may involve a more comprehensive modulation of the underlying sensory processing, and not only within higher-level valuation circuitry. One caveat, which is shared with research on other sensory modalities, is that although thinking in terms of one ascending “sensory” system and one descending “modulatory” system is useful, e.g., for forming testable research questions, it may be too simplistic. It has been suggested that, instead of functioning as separate systems where one can influence the other, it may be more accurate to consider this as one recurring sensory processing system with several integrative components, such as feed-forward signaling, feedback loops, influences from the “early” sensory processing of other modalities, and influences from more abstract cognitive and affective information (Kinchla and Wolfe, 1979; Finkel and Edelman, 1989; Ullman, 1995; Siegel et al., 2000; O’Reilly et al., 2013).

## Neurochemical Basis of the Motivation for Affiliative Touch

In affiliative interactions such as rough-and-tumble play, where touch has stimulatory or arousing behavioral or physiological effects (Feldman et al., 2010a; Gordon et al., 2010), the touch behaviors involved are typically different from interactions where the intent is soothing, consolidation or relaxation (Holt-Lunstad et al., 2008). These touch activities are likely driven by different motivational modes, depending on the individual’s underlying needs. There is evidence that primates and rodents frequently engage in soothing and soft touch activities, like social grooming and huddling, but rarely in rough-and-tumble play, when they are distressed or in homeostatic imbalance (For review, see Løseth et al., 2014). However, while stimulatory and soothing touch may generally be differentiated by their stimulus characteristics, the actual arousing or relaxing effects of a given kind of touch depends on the appraised meaning (Ellingsen, 2015). For example, although a gentle caress can be soothing in one context, it can be sexually arousing in a different context. Furthermore, a caress can arouse negative affect and withdrawal if coming from an unwanted person (Major, 1981).

### State-Dependent μ-Opioid Modulation of Affiliative Touch

The μμ-opioid receptor (MOR) system has a multi-faceted role in reward, both social (Machin and Dunbar, 2011; Chelnokova et al., 2014) and non-social (Drewnowski et al., 1995; Yeomans and Gray, 1997). As is well known, MOR activation promotes relief of negative affect (Hsu et al., 2013) – e.g., drugs that activate MOR in humans have potent analgesic effects (Zubieta et al., 2001). Moreover, opioids have an inhibitory effect on Hypothalamic–Pituitary–Adrenal (HPA) axis responses to environmental stress (Kreek, 1996; Wand et al., 2002). Furthermore, MOR promotes motivation for (Mahler and Berridge, 2012) and enjoyment of (Pecina and Berridge, 2005) appetitive reward. A wealth of studies using pharmacological manipulation of the MOR system in a variety of mammalian taxa have demonstrated a key role of the MOR system for affiliative touch behaviors, such as social grooming (Keverne et al., 1989; Machin and Dunbar, 2011), social play (Panksepp and Bishop, 1981; Vanderschuren et al., 1995), and huddling (Shapiro et al., 1989; Dunbar, 2010).

Notably, the directionality of MOR agonism and antagonism effects on affiliative touch behaviors has been diverging into two opposing “camps” – 1) studies of primates and infant rodents indicating that enhanced MOR signaling *reduces* affiliative touch behaviors, and 2) studies of adolescent and adult rodents indicating that enhanced MOR signaling *increases* affiliative touch behaviors. We recently proposed the State-dependent μ-Opioid Modulation of Social Motivation (SOMSoM) model as a resolution to this apparent paradox, in that, instead of reflecting a fundamental species-related difference in MOR function per se, these differences may instead be due to consistent differences in the animals’ motivational state during the experimental tasks (Løseth et al., 2014). Most of these studies made use of some variant of a “social relief paradigm”, where the animal was separated for a certain amount of time before reunited with its peers. Since primates and infant rodents rely on close social bonds with others for survival and protection, they are often very distressed by social separation (Panksepp et al., 1978). Adolescent and adult rodents on the other hand, are not as reliant on social support for survival or coping with stress, and typically form more transient bonds for mating and parenting. Thus, they are considerably less distressed by the social separation that is inherent in the majority of these studies (Nelson and Panksepp, 1998; van den Berg et al., 1999). Consequently, while socially isolated primates and infant rodents may be distressed and thus highly motivated for seeking relief and safety through social contact, adult and adolescent rodents may have less need for relief and thus more motivation for social exploration. The SOMSoM model proposes that during negative affective states, animals seek out affiliative touch interactions primarily for comfort and relief of negative emotion. By providing relief from distress, MOR activation by social contact or pharmacological stimulation therefore reduces contact seeking, while disruption of MOR signaling intensifies contact seeking. However, when the animal is in emotional equilibrium, social interactions are instead sought out for exploration, joy, and mating, which is also promoted by MOR (Løseth et al., 2014). During this motivational state, pharmacological stimulation of MOR signaling increases, while disruption of MOR signaling reduces, contact seeking and behaviors such as play.

Touch plays a central role in these interactions, and a body of behavioral research indicates a specific role for affiliative touch in health and wellbeing (for review, see Walker and McGlone, 2013). However, the social interactions in these studies are always happening in a rich multisensory context. An important challenge for future studies is therefore to disentangle the role of the MOR system in touch specifically.

### Oxytocin, Social Affiliation, and Affective Touch

The neuropeptide oxytocin also plays a central role in social affiliation and attachment in mammals (Tops et al., 2007; Feldman, 2012). Differences in oxytocin receptor distribution in limbic brain areas across rodent species reflect differences in social organization and bond formation (Young et al., 2011). The monogamous prairie vole has higher densities of oxytocin and vasopressin receptors in the ventral striatum than the closely related, but promiscuous, montane and meadow voles (Ross et al., 2009). Furthermore, the blockade of mesolimbic oxytocin signaling in prairie voles prevents both maternal behavior (Cho et al., 1999; Olazabal and Young, 2006) and the formation of long-term pair bonds (Insel and Hulihan, 1995; Cho et al., 1999; Ferguson et al., 2000). Oxytocin is also involved in a range of social and emotional processing in humans (Bartz et al., 2011; Leknes et al., 2013; Ellingsen et al., 2014), and has anxiolytic effects (Heinrichs et al., 2003; Kirsch et al., 2005), enhance parasympathetic responses (Gamer and Buchel, 2012), and increase heart rate variability – indicating increased vagal control (Kemp et al., 2012). Similar to the MOR system, oxytocin is associated with promoting social approach both for appetitive social reward (Panksepp et al., 1997; Nakajima et al., 2014), and relief of negative affect (Campbell, 2008; Bosch, 2011). Specific affiliative behavior such as social grooming (Drago et al., 1986; Pedersen et al., 1988; Witt et al., 1992; Francis et al., 2000; Champagne et al., 2001; Champagne, 2008) and maternal nurturing (Pedersen and Prange, 1979; Pedersen et al., 1982; Bosch, 2011) has been associated with oxytocin.

Several studies indicate that oxytocin suppresses the activity of the stress-induced HPA axis. In humans, oxytocin reduces the release of adrenocorticotropic hormone (ACTH; Chiodera and Coiro, 1987) and cortisol (Legros et al., 1988) in response to stressful stimuli. In rats, central blockade of oxytocin increases basal and stress-induced release of ACTH and corticosterone (Neumann et al., 2000b). However, a study using local injection of an oxytocin antagonist indicates differential effects on stress responses depending on the brain site. Local blockade in the Paraventricular Nuclei (PVN) led to increased basal ACTH, but reduced stress-induced release of ACTH, perhaps because of the increased baseline. On the other hand, injections in the amygdala and the medio-lateral septum, which projects directly and indirectly to the PVN, did not alter basal ACTH levels, but reduced stress-induced ACTH (Neumann et al., 2000a). Because of the effect of oxytocin on both stress regulation and social bonding, it has been suggested that the soothing and anxiolytic effects of stroking touch in mammals is mediated by oxytocin (Uvnas-Moberg et al., 2014).

In contrast to in rodent literature, relatively few studies have employed pharmacological modulation of oxytocin in primates. A recent study investigated pair-bonding in marmoset monkeys, and found that huddling behavior was increased by the administration of an oxytocin receptor agonist, but reduced by an oxytocin antagonist (Smith et al., 2010). Another study found that, in squirrel monkeys, intranasal oxytocin dampened the increases of blood plasma ACTH in response to (stressful) social isolation. However, plasma levels of cortisol were not affected (Parker et al., 2005), and, since behavioral changes were not assessed, it is difficult to directly relate this finding to affiliative touch behavior.

Primate studies investigating peripheral levels of oxytocin during social interactions provide indirect evidence for an involvement of oxytocin in affiliative touch behavior (although whether peripheral OT levels give an indication of central OT levels is as yet unclear – see Jokinen et al., 2012; Neumann and Landgraf, 2012; Kagerbauer et al., 2013). In rhesus monkeys, engagement in social grooming activities correlates positively with plasma (Maestripieri et al., 2009) and cerebrospinal fluid (Winslow et al., 2003) levels of oxytocin. In wild chimpanzees, increased urinary levels of oxytocin has been reported to follow grooming events, which is mediated by bond strength between the grooming partners, specifically grooming interactions between animals with closer social bonds showed larger increases in urinary oxytocin (Crockford et al., 2013). Another study found no relationship between plasma oxytocin and social behavior in free-ranging macaques (Schwandt et al., 2007). A recent study on pair bonding in cotton-top tamarins reported that inter-individual levels of urinary oxytocin co-varied closely with grooming and mutual contact in females and with sexual behavior in males (Snowdon et al., 2010). Moreover, one study reported higher urinary levels of oxytocin during social contact than during social isolation (Seltzer and Ziegler, 2007). Together, these studies are in line with a notion that oxytocin release is associated with the relief of negative states induced by social isolation or rejection, and that low levels of oxytocin may promote seeking of social support (Panksepp et al., 1997; Tops et al., 2007). It has been proposed that oxytocin release in social interaction may involve two “phases” – first, a social-salience related release during motivation for approach, and second – if leading to physical affiliative contact – an anti-stress related release (Uvnas-Moberg et al., 2014). This model is derived from the reports of oxytocin release in dogs and dog-owners, first in response to auditory and visual cues that the other “individual” is nearby, and then again when the owner strokes and caresses the dog, together with reductions in plasma cortisol (Miller et al., 2009; Handlin et al., 2011; Beetz et al., 2012; Rehn et al., 2014).

Similar to the effects of MOR system manipulations, the behavioral effects of oxytocin administration seem to vary across contexts and affective states (Bartz et al., 2011). In rodents, oxytocin is associated with both protective behavior toward pups and aggression against intruders (Campbell, 2008). Intranasal oxytocin in humans increases the recognition of both positive (Unkelbach et al., 2008; Marsh et al., 2010) and negative emotions (Bartz et al., 2010; Fischer-Shofty et al., 2010; Leknes et al., 2013), and increases empathizing and cooperation with in-group members, but may increase aggression toward threatening out-group members (De Dreu and Kret, 2015). We recently found that oxytocin promotes a social-touch induced “sharpening” of social impressions of others, relative to non-social touch (Ellingsen et al., 2014). Nevertheless, a single dose (40IU) of oxytocin did not affect the pleasantness or intensity of the actual touch experience. In contrast, a recent study found that, in a group of heterosexual men, intranasal oxytocin increased the pleasantness of sensual caresses specifically when they believed that a woman was touching (Scheele et al., 2014). However, oxytocin had no effect on touch pleasantness when the participants believed the caresser was a man, further highlighting the importance of multisensory context in oxytocin functioning. A popular hypothesis about central oxytocin functioning is that oxytocin may promote social approach behavior (in both positive and negative contexts), and inhibit social avoidance (Kemp and Guastella, 2010, 2011; Clark et al., 2013). However, while many of the studies using intranasal oxytocin in humans involve an experimental manipulation of context, they rarely assess – or manipulate – more profound changes in motivational or homeostatic state. Thus, it is not well known to what degree oxytocinergic modulation of social approach and avoidance in humans depends on the individual’s initial state. Interestingly, it has recently been suggested that oxytocin might promote approach behavior more strongly in novel contexts compared to familiar contexts (Tops et al., 2014). One study found that participants’ salivary oxytocin, during anticipation of a cognitive task, was positively correlated with state trust at the initial session, but negatively correlated with trust in the subsequent session, when they were familiar with the task (Tops et al., 2013). Similarly, another study reported that intranasal oxytocin increased the expression of affiliation in a clinical interview for depression, during an initial visit but not during a follow-up visit (Brune et al., 2015).

A series of studies assessing endogenous peripheral levels of oxytocin suggest a role of oxytocin in human affiliative touch (Lupoli et al., 2001; Matthiesen et al., 2001; Uvnas-Moberg, 2004; Light et al., 2005), although the exact mechanisms are unclear (Feldman, 2012). One study found that plasma oxytocin levels in mothers during pregnancy and the early postpartum period predicted maternal bonding behaviors such as eye gaze, high-pitched vocalizations and affectionate touch directed at the infant (Feldman et al., 2007). Another study reported that higher plasma levels of oxytocin correlated with more frequent infant-directed stimulatory touch by first-time fathers, but with more frequent affectionate touch (e.g., hugging, kissing, and stroking) by first-time mothers (Gordon et al., 2010). Furthermore, one study found that couples who were instructed to perform 30 minutes of reciprocal “warm, sensual” touch on their partner’s neck, shoulders, and hands, three days/week for 4 weeks, had increased post-intervention levels of salivary oxytocin, as well as reductions in stress-responsive markers such as blood pressure, plasma cortisol, and alpha amylase, compared to a control group (Holt-Lunstad et al., 2008). Unfortunately, this literature commonly quantified oxytocin in plasma using methods in which the validity has been questioned, and results may reflect non-oxytocin substances (McCullough et al., 2013; Christensen et al., 2014). Perhaps for this reason, along with potentially fine-grained variations in context and motivational state across studies, overall findings of touch-induced release of peripheral oxytocin in humans are inconsistent. While some studies have found peripheral oxytocin release in response to touch (Light et al., 2000; Odendaal and Meintjes, 2003; Light et al., 2005; Holt-Lunstad et al., 2008), others have found no effect (Turner et al., 1999; Heinrichs et al., 2001; Wikstrom et al., 2003; Grewen et al., 2005; Ditzen et al., 2007). Moreover, methodological limitations like the lack of useful oxytocin antagonists for human testing, as well as the current inability to assess oxytocin release in the human brain, limits the understanding of the functional neurobiology of oxytocin in humans. Finally, it is important to note that, like the MOR system, many of the functions of central oxytocin are not restricted to the social domain, but instead may reflect more fundamental mechanisms involved in generalized processing of salience, motivation, anxiety, and stress regulation (Churchland and Winkielman, 2012; Harari-Dahan and Bernstein, 2014).

### Contribution of Other Neurotransmitters

In addition to MOR and oxytocin, neurotransmitters such as vasopressin (Winslow et al., 1993; Panksepp et al., 1997), serotonin (Insel and Winslow, 1998; ?), cannabinoids (Trezza and Vanderschuren, 2008a,b; Trezza et al., 2012) and dopamine (Champagne et al., 2004) modulate social touch behaviors in mammals. For example, tickling – an activity primarily associated with social play – increases NAc dopamine signaling in rats (Maruyama et al., 2012; Hori et al., 2013). In humans, massage therapy increases urinary dopamine and serotonin, and reduces urinary and salivary cortisol (as reviewed by Field et al., 2005). It is, however, unknown whether such peripheral assessment reflects concentrations of these neurotransmitters in the brain.

These neurotransmitter systems likely interact with MOR and oxytocin processing in key brain regions involved in social and emotional processing (Hagelberg et al., 2002; Liu and Wang, 2003; Depue and Morrone-Strupinsky, 2005a; Lintas et al., 2011; Colasanti et al., 2012; Tops et al., 2014). Understanding the nature of these interactions is an important challenge for future studies (Weisman and Feldman, 2013). Furthermore, in the periphery, oxytocin interacts with other hormones to affect behavior. For example, it has been reported that intranasal oxytocin increases human fathers’ expression of eye gaze and affectionate touch toward their infants, but only in those whose plasma testosterone levels also increase after the oxytocin administration (Weisman et al., 2014).

## Conclusion

Although touch can be a source of safety, comfort, relief, and pleasure, this effect is likely confined to instances where contextual cues are affectively congruent with affiliative touch, e.g., when the other individual is friendly, has good intentions, and the touch is socioculturally appropriate. When touch occurs in combination with contextual cues indicating that the touch is undesirable, or is associated with danger, the same touch stimulus may instead be appraised as unpleasant or disgusting and promote avoidance. It is not yet known whether there are aspects of touch that are inherently positive, or if the hedonic value of all kinds of touch is dependent on context or internal state. Future research is needed to determine the flexibility and boundaries of bottom-up versus top-down influences on touch. Although relatively few, the existing studies on the neurobiological underpinnings of top-down modulation of affective touch indicate involvement of modulatory prefrontal and subcortical circuitry key to valuation, cross-sensory integration, and the construction of meaning (Ellingsen et al., 2013; Ellingsen et al., 2015). These studies also suggest that somatosensory processing in circuitry traditionally considered part of a “bottom-up” pathway can be modulated by expectations and contextual cues informative of the hedonic value of touch. This mechanism bears similarity to that involved in placebo improvement of negative hedonic experiences, such as pain (Scott et al., 2007; Eippert et al., 2009a; Benedetti, 2014). μ-opioids and oxytocin are two of the neurotransmitters that have been most extensively studied in relation to affiliative touch. Pharmacological manipulation of μ-opioid processing can dramatically influence touch behavior in mammals, but the directions of the effects seems to depend on motivational state. Whereas MOR antagonism increases social contact seeking when the animal is distressed, it tends to decrease contact seeking when the animal is in a non-stressed state, especially in animals that rely on social relationships for emotion regulation, such as primates and infant rodents (Panksepp et al., 1978). This may reflect a bimodal role of opioids in both comfort seeking and exploration for social reward, mirroring the dual effects of MOR in pain relief and pleasure (Løseth et al., 2014). The role of oxytocin in affiliative touch, and in social interactions in general, is similarly dependent on context. One line of research indicates that oxytocin may either increase the salience of socially relevant cues, or promote approach behavior in general (Shamay-Tsoory et al., 2009; Kemp and Guastella, 2010, 2011). Another line of research indicates an anxiolytic and stress-reducing effect of oxytocin, and it has been hypothesized to account for the relaxing and soothing effects of touch (Churchland and Winkielman, 2012). Unfortunately, most of the studies investigating the effects of pharmacological manipulation of μ-opioids and oxytocin systems on social touch behaviors do not give information about the contribution of touch relative to other sensory modalities. The same issue applies to investigations of the effects of interpersonal touch in naturalistic settings, where touch is part of a complex multisensory interaction. A future challenge is thus to disentangle the specific role of touch in relation to other sensory modalities, and how touch is integrated with other sensory signals. Isolating the specific role of touch in social interactions, while still keeping a certain level of ecological validity is particularly challenging, and poses an important task for future research.

## Author Contributions

DE prepared the manuscript. DE, SL, GL, JW, and HO revised the manuscript into its finalized form.

## Conflict of Interest Statement

The authors declare that the research was conducted in the absence of any commercial or financial relationships that could be construed as a potential conflict of interest.
